# Vaccination induced antibodies to recombinant avian influenza A virus M2 protein or synthetic M2e peptide do not bind to the M2 protein on the virus or virus infected cells

**DOI:** 10.1186/1743-422X-10-206

**Published:** 2013-06-24

**Authors:** Willem J C Swinkels, Jeroen Hoeboer, Reina Sikkema, Lonneke Vervelde, Ad P Koets

**Affiliations:** 1 Department of Farm Animal Health, Faculty of Veterinary Medicine, Utrecht University, Yalelaan 1, 3584, Utrecht, CL, The Netherlands; 2 Department of Infectious Diseases and Immunology, Faculty of Veterinary Medicine, Utrecht University, Yalelaan 1, 3584, Utrecht, CL, The Netherlands; 3 The Roslin Institute, Edinburgh University, Edinburgh, UK

**Keywords:** Influenza, Chicken, M2, Peptide Vaccination

## Abstract

**Background:**

Influenza viruses are characterized by their highly variable surface proteins HA and NA. The third surface protein M2 is a nearly invariant protein in all Influenza A strains. Despite extensive studies in other animal models, this study is the first to describe the use of recombinant M2 protein and a peptide coding for the extracellular part of the M2 protein (M2e) to vaccinate poultry.

**Methods:**

Four groups of layer chickens received a prime-boost vaccination with recombinant M2 protein, M2e, a tetrameric construct from M2e peptide bound to streptavidin and a control tetrameric construct formulated with Stimune adjuvant.

**Results:**

We determined the M2-specific antibody (Ab) responses in the serum before vaccination, three weeks after vaccination and two weeks after booster, at days 21, 42 and 56 of age. The group vaccinated with the M2 protein in combination with Stimune adjuvant showed a significant Ab response to the complete M2 protein as compared to the other groups. In addition an increased Ab response to M2e peptide was found in the group vaccinated with the M2e tetrameric construct. None of the vaccinated animals showed seroconversion to AI in a commercial ELISA. Finally no Ab’s were found that bound to M2 expressed on *in vitro* AI infected MDCK cells.

**Conclusion:**

Although Ab’s are formed against the M2 protein and to Streptavidin bound M2e peptide in a tetrameric conformation these Ab’s do not recognize of M2 on the virus or on infected cells.

## Background

Avian influenza virus (AIV) is mostly classified by its surface antigens hemagglutinin (HA) and neuraminidase (NA). Antigenic drift and antigenic shift of these two immunodominant surface proteins makes it difficult to construct a universal vaccine [1]. The matrix 2 (M2) surface protein forms an ion channel and is needed for the release of viral ribonucleoprotein (vRNP) from the matrix protein 1 (M1) into the cell cytoplasm [2]. The conserved nature of the extracellular domain of M2 (M2e) makes it an attractive target for developing a vaccine to a broad spectrum of influenza A viruses [3-7].

A natural infection renders a limited antibody response against M2e in both men and mice, probably due to its low copy number on virions, its small size (97 amino acids) and the small size of the extracellular domain (23 amino acids without Met1) [8-10]. Experiments have shown that vaccination of mice with an M2-hepatitis B virus core (HBc) fusion protein can generate antibodies, that after serum transfer protect against a lethal virus challenge [5]. Wu et al. found that M2e alone was a poor immunogen which did not elicit a significant immune response in mice, while combined with aluminim or Freund adjuvant the peptide was immunogenic and vaccination protected against lethal dose of influenza virus challenge [11]. Current used vaccines include peptide carrier conjugates [6], baculovirus expressed M2 [12], M2 fusion proteins [3], multiple antigenic peptides [13], and M DNA vaccine [14,15]. M2e conjugated influenza vaccines have been shown to be highly immunogenic in mice, ferrets and rhesus monkeys and protective against homologous and heterologous challenge with influenza A virus [6,12,13,16,17]. Most M2 vaccines have been tested in mice while few experiments have been performed in other species. In pigs, contrary to mice a natural host, vaccination with an M2e fusion protein has been shown to exacerbate disease symptoms after challenge [18]; similar to what was found when inactivated SIV was used in a heterologous challenge model [19]. Recently it was shown that SIV vaccine associated respiratory problems could be decreased when recombinant M2 was added to the vaccine. However, again M2 alone did not reduce virus shedding [20]. These data suggest that for each species it is necessary to find out whether vaccination with M2 protein or subunits of this protein has a potential. In chickens Nayak et al. tested recombinant NDV vectors that expressed each of the three surface proteins of high pathogenic AIV. They found no indication for M2 to be immunogenic or protective [21]. Zhang et al. used a plasmid coding for an M2 protein of which the transmembrane region was deleted. They found that chicken C3d enhanced the humoral immunity against AIV M2 protein, be it with a poor protection ratio [22]. In contrast, Layton et al. (2009) showed that Salmonella vectored vaccines expressing a M2e epitope in association with CD154 are effective at protecting chickens against LPAI, but not against HPAI [23].

This article describes for the first time the use of a full length M2 protein and synthetic M2e peptide to vaccinate chickens, a natural host of avian influenza. We report a study in which we vaccinated chickens with Stimune adjuvanted full length M2 protein, M2e peptide and Streptavidin bound M2e peptide in a tetrameric conformation.

## Results

### M2 expression

The identity of the cloned genes was confirmed by PCR and gene sequencing. A band with approximately 15 kDa was observed after induction of the His-tagged M2, which was confirmed by Western-blot analysis using a commercial rabbit anti-M2 antibody. After induction of the MBP-tagged gene, a band with approximately 55 kDa was observed, corresponding with the correct size of MBP (42 kDa) and M2 (15 kDa). The induction of both constructs showed to be disadvantageous for bacterial growth as compared to bacteria with no construct in their plasmid. Due to the decrease of bacterial growth after induction, the yield of M2 protein was low (< 0.5 mg/l induced medium).

### Antibody detection

**Figure 1:**
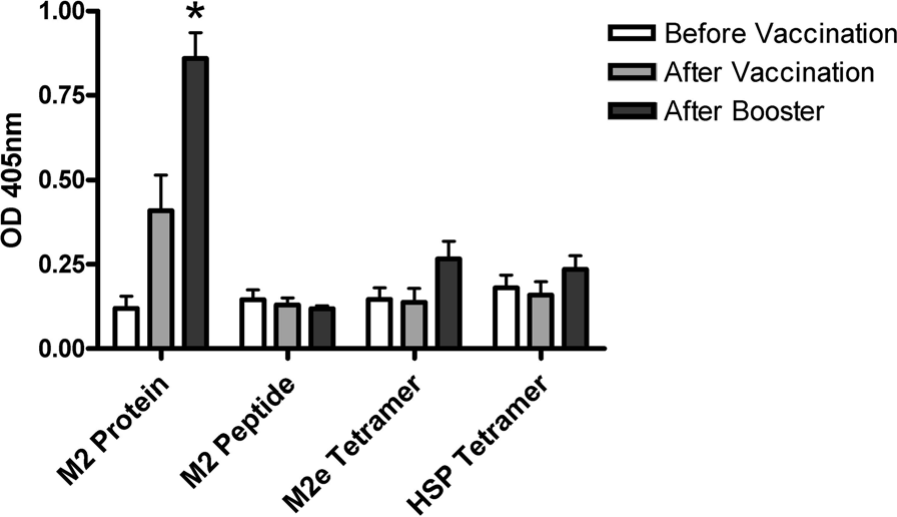
**MBP-M2 protein specific antibodies in chicken sera obtained at the time of vaccination, three weeks after vaccination and two weeks after booster vaccination were determined by ELISA.** The ELISA plate was coated with 0.5 μg/well affinity purified *E. coli* recombinant MBP-M2 protein. Vaccination groups are indicated on the X-axis by antigen present in the vaccine (M2 Protein = *E.coli* recombinant his-tagged M2 protein; M2e Peptide = synthetic M2e peptide, M2e Tetramer = synthetic biotinylated M2e peptide conjugated with Streptavidin, HSP70 Tetramer = synthetic biotinylated HSP70 control peptide conjugated with Streptavidin). Average OD 405 nm value +/− SEM from five animals in each group is shown. * = significant difference between indicated group and the same group before vaccination.

To test if antibodies from the sera recognized the M2 protein, an ELISA using MBP-M2 protein was performed (Figure 1). Antibody responses were only detected to MBP-M2 protein after booster vaccination with full length His-M2 protein. In the peptide immunized-groups no response to the full length MBP-M2 protein was found in the ELISA.

**Figure 2:**
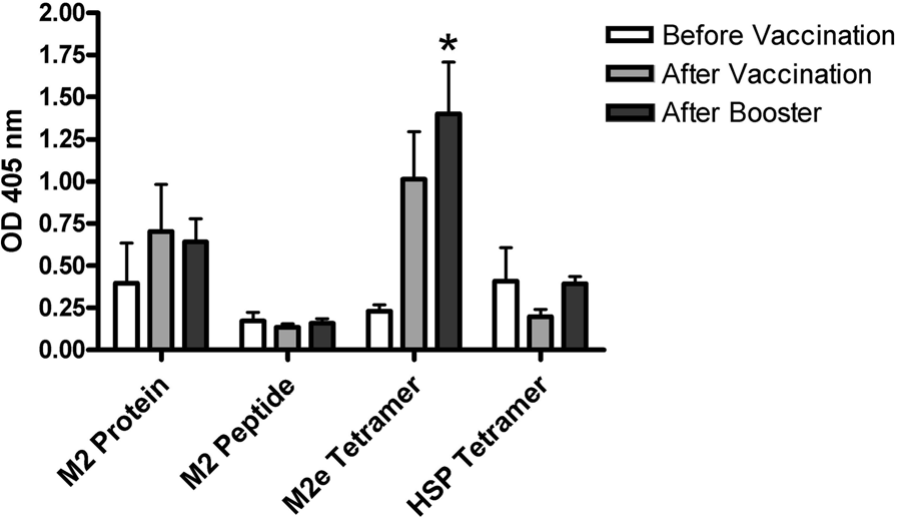
**M2e peptide specific antibodies from sera obtained at the time of vaccination, three weeks after vaccination and two weeks after booster were determined by ELISA.** The ELISA plate was coated with 0.5 μg/well synthetic M2e peptide. Vaccination groups are indicated on the X-axis by immunogen present in the vaccine (M2 Protein = *E. coli* recombinant his-tagged M2 protein; M2e Peptide = synthetic M2e peptide, M2e Tetramer = synthetic biotinylated M2e peptide conjugated with Streptavidin, HSP70 Tetramer = synthetic biotinylated HSP70 control peptide conjugated with Streptavidin). Average OD405 nm value +/− SEM from five animals in each group is shown. * = significant difference between indicated group and the same group before vaccination.

We also investigated if our vaccination provoked an antibody response against the M2e peptide (Figure 2). After vaccination with the full length His-M2 protein, the M2e peptide or the HSP-tetrameric construct, no significant antibody responses were detected to the M2e peptide. Immunization with the M2e peptide tetrameric construct showed a significant antibody response to M2e peptide after booster.

**Figure 3:**
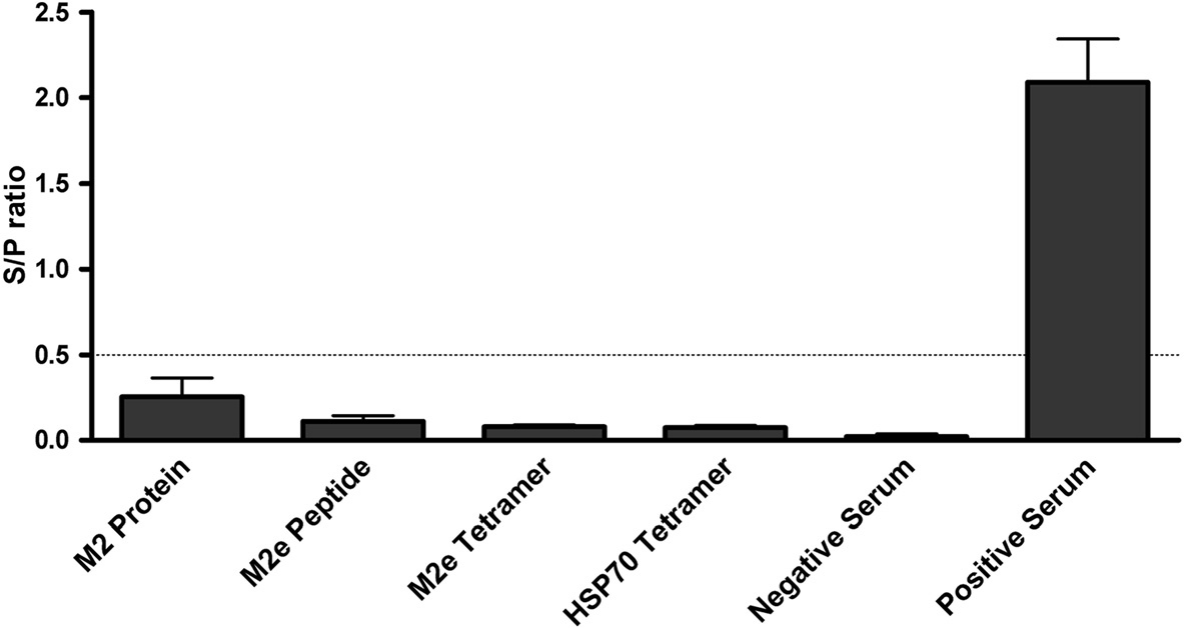
**Measurement of antibodies to avian influenza in serum with the IDEXX AI Flockcheck Test (IDEXX).** Vaccination groups are indicated on the X-axis by immunogen present in the vaccine (M2 Protein = *E. coli* recombinant his-tagged M2 protein; M2e Peptide = synthetic M2e peptide, M2e Tetramer = synthetic biotinylated M2e peptide conjugated with Streptavidin, HSP70 Tetramer = synthetic biotinylated HSP70 control peptide conjugated with Streptavidin). The average value of the sample-negative/positive–negative (S/P) ratio +/− SEM from five animals in each group is shown. In addition the average value of the sample-negative/positive–negative (S/P) ratio +/− SEM from triplicates of a negative and positive reference sample from naive and H9N2 vaccinated chickens is shown (GD Deventer, The Netherlands). The threshold value of 0.5 for a positive S/P ratio as per manufacturer instruction is depicted as a dotted line.

To test whether the antibodies recognized AIV we used a commercial avian influenza ELISA. In Figure 3 the Sample/Negative (S/N) ratio is depicted. When the outcome of this ratio is above 0.5, the sample is regarded as AI positive. As an extra control of the kit we used sera from five control animals and five animals which showed seroconversion in the AI Flockcheck test after H9N2 A Chicken/Saudi Arabia/SP02525/3AAV/2000 influenza challenge. In this test none of the vaccinated groups had a positive result. The negative control sera showed no result above the threshold, whereas the positive control sera showed a positive S/P ratio in the ELISA.

**Figure 4:**
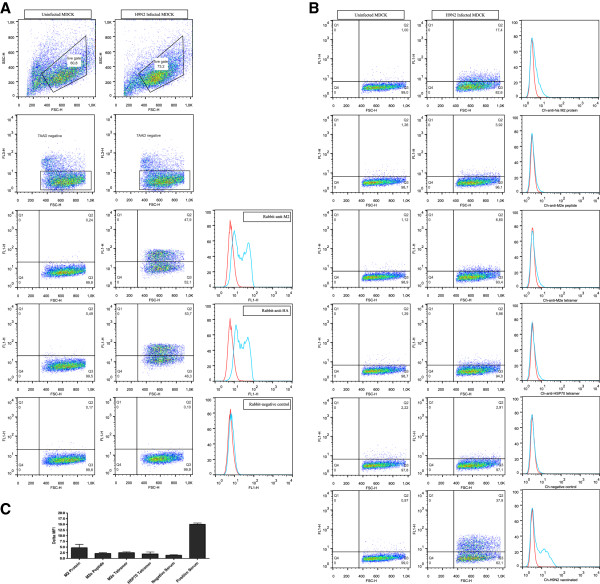
**Antibody binding to H9N2 infected MDCK cells. (A)** Representative dot plots of uninfected MDCK cells (left column), H9N2 Influenza infected MDCK (middle column) and overlays of specific antibody responses (right panels) are depicted. Top panels shown FSC-SSC dot plots, second line of panels indicate 7AAD live/dead discrimination in FL3 channel. Third row shows staining of live uninfected (left) and H9N2 infected (middle) MDCK cells with rabbit-anti-M2 antibody and the respective overlay (right panel). Fourth row shows staining of live uninfected (left) and H9N2 infected (middle) MDCK cells with rabbit-anti-HA antibody and the respective overlay (right panel). Fifth row shows staining of live uninfected (left) and H9N2 infected (middle) MDCK cells with naïve rabbit control antibody and the respective overlay (right panel). **(B)** Representative dot plots specific antibody responses of one of five vaccinated chickens to uninfected MDCK cells (left column), H9N2 Influenza infected MDCK (middle column) and overlays of (right panels) are depicted for each indicated treatment group (top to bottom: his-M2 protein, M2e peptide, M2e tetramer and HSP70 tetramer), followed by negative and H9N2 vaccinated positive chicken reference serum. **(C)** The average value of the mean fluorescence intensity (MFI) of the H9N2 infected MDCK cells minus the MFI of the uninfected MDCK cells (as depicted in **(B)**) +/− SEM from five animals in each treatment group (indicated on the X-axis as M2 protein, M2e Peptide, M2e Tetramer and HSP70 Tetramer respectively) as well as triplicates +/− SEM of a negative and positive reference sample from naive and H9N2 vaccinated chickens (GD Deventer, The Netherlands) is shown as a Delta MFI. No significant differences between treatment groups were observed.

The amount of M2 protein on infected cells is much higher than the amount of this protein on virus [24,25]. We infected MDCK cells and tested whether the antibodies induced by the different vaccines were able to bind to these infected cells (Figure 4). When gated on live cells, sera from all groups had a significant higher amount of binding to infected cells compared to the non infected cells. Mean fluorescence intensity (MFI) from the non infected cells was subtracted from the MFI of the infected cells. The same negative and positive chicken sera were used as in the ELISA. Controls with commercial antibodies showed that infected MDCK expressed M2 and H9. Serum from chickens obtained two weeks after infection with influenza showed binding to infected MDCK. No significant statistical differences however were found when the groups vaccinated with His-M2 protein, M2e peptide or M2e peptide tetrameric construct were compared to the group which got the mock vaccine with the HSP tetrameric construct.

## Discussion

The conserved nature of the influenza M2 protein makes it a promising candidate for a universal influenza vaccine. Most vaccine studies on M2 are performed in mice, while the few experiments described for chicken show variable outcome [21-23]. Our article describes for the first time the use of a full length His-M2 protein and an M2e peptide to vaccinate chicken.

The M2 protein is a proton channel and the integration of such a protein in the membrane might lead to membrane leakage and loss of cell viability. In our prokaryotic expression system, expression of His-M2 and MBP-M2 form led to a decrease of cell growth which might be indicative for the bioactivity M2. Though in low yield, as was previously described by Frace et al. [16], we were able to produce the complete M2 protein. His-M2 was used to investigate if the M2 protein was immunogenic following vaccination of poultry. Apart from the protein, we used a peptide coding for M2e. Because of the known low immunogenicity of the single peptide we also made a more immunogenic tetrameric construct by binding the biotinilated peptide to streptavidin. As a construct control we made a mock construct with a non influenza related peptide derived from a HSP70 sequence.

Sera obtained after a vaccination with the full length His-M2 protein showed a positive reaction in our MBP-M2 ELISA. The single M2e peptide showed not to be immunogenic, while in tetrameric conformation it proved to be able to induce a serum antibody response.

In our vaccinated animals no seropositivity was found using a commercial ELISA kit, which means that antibodies against the vaccines do not recognize influenza virus as coated on the plate. It is not known how sensitive this commercial test is for detecting M2-specific antibodies. Specific chicken antisera to HA, NP and M2 were not available to address this question. It is quite likely that the test has a higher sensitivity in detecting other influenza specific antibodies to more abundant influenza antigens such as HA and NP over M2*.* Jegerlehner et al. and Kaiser et al. stated that M2 antibodies do not bind to influenza virus nor neutralize virus infection, but only bind to the protein on infected cells to promote their clearance [26,27]. Since antibodies against the M2 protein might play an important role in directing effector cells to their targets and the amount of M2 is much higher on infected cells than on virions, we extended our readout from an ELISA system to a flow cytometry based protocol with infected MDCK cells [28]. After infection, the MDCK cells express the viral proteins, including M2, on their surface as was shown by fluorescent labeling. In sera from all groups, obtained two weeks after avian influenza infection, binding to infected cells MDCK was observed, but when compared to the HSP group however, none of the groups showed a significant difference. This makes binding to infected cells not likely to be specific binding to the native tetrameric form of the M2 protein. Antibodies as found by ELISA were not able to bind M2 on infected cells and therefore recognize only the monomeric but not the tetrameric native form of the protein.

A synthetic M2e peptide coupled to KLH [29], and even adjuvanted synthetic M2e peptide without carrier proteins were able to generate M2e-specific immune responses in mice [11]. Combined with the positive vaccination experiments in chicken [22,23] this led to the expectation that M2 protein and M2e peptide vaccination would work in chicken as well. Our study shows that it is possible in chicken to raise antibodies against a bacterial expressed avian influenza M2 protein or streptavidin bound M2e peptide in a tetrameric conformation. Antibodies raised to the recombinant M2 protein have a tendency to react to M2e peptide however this effect was not significant probably due to the fact that more epitopes are present in M2 protein. Antibodies generated to M2e peptide-tetramer showed limited binding in the M2 protein ELISA. The M2e epitope density in the MBP-M2 coated ELISA system may be limiting for M2e-peptide generated antibodies as the MBP constitutes a 42 kD tag per M2. Though our vaccinations proved to be immunogenic, this did not lead to statistically significant recognition of native M2 by chicken serum, be it on the virus or on infected cells. The problem with extrapolating results for M2 vaccines from mice to other animals was shown by Fu et al. in an experiment in which they compared the immunogenicity of a vaccine in mice and rhesus monkeys [30]. Experiments with vaccines based on M2 protein in chicken and pig, both natural hosts of influenza, have not been as successful as in mice, which forces us to rethink vaccine composition. Current poultry vaccines consist of inactivated whole virus in oil emulsion, live attenuated viruses, recombinant viruses or immune-complexed viruses while research focuses on the development of subunit vaccines [31]. The peptide vaccines, especially the ones based on M2e, are poorly immunogenic and need a strong adjuvant. In poultry, so far to our knowledge no synthetic peptide vaccines have been described. Increasing immunogenicity by coupling the peptide to KLH [6] or using cytokines as adjuvants [31] might lead to better responses and functional vaccines.

## Conclusion

Our data suggest that for each species it is necessary to find out whether vaccination with M2 protein or subunits of this protein has a potential. In chickens, a natural host of avian influenza, a vaccination with the full length M2 protein or synthetic M2e peptide in a tetrameric conformation proved to be immunogenic but the induced antibodies did not recognize M2 on the virus or on infected cells. This makes our components in their present form not applicable as single unit vaccines, but as an additional subunit in a vaccine containing more peptides based on viral proteins such as HA and NA, M2(e) might still be able to play a role in protection against influenza viruses.

## Methods

### Chickens

Day-old Lohmann Brown Light layer chickens (Verbeek) were tagged and housed with saw dust bedding in the faculty animal housing facilities. Chicks were fed *ad libitum* with free access to drinking water. The experiment was carried out in accordance with the Dutch regulation on experimental animals and approved by the Animal Experiment Committee of Utrecht University.

### M2 protein constructs

The LPAI strain: A/chicken/Italy/1067/99 (H7N1) was used as cDNA source; kind gift of Dr I. Capua (Istituto Zooprofilattico Sperimentale delle Venezie, Italy). The M2 gene is the product of a spliced M gene. M2 (aa10-97) sequence lacking the first 26 basepairs was cloned this cDNA into a Pet100 plasmid (Invitrogen). With an elongated primer complete M2 was cloned into a Pet100 plasmid. For MBP-M2 protein expression, complete M2 with restriction sites for EcoRV and BamHI was cloned into the pMAL-p5X vector (New England Biolabs). All primers are displayed in Table 1. Identity of cDNA and cloned PCR products were checked by sequencing.

**Table 1:** Primers used to amplify the M2 sequences

**Primer name**	**Primer sequence**
M2(aa10-97) F	CACCTACCAGAAACGGATGGGAGTGCAAA
M2(aa10-97) R	TTACTCCAGCTCTATGTTGACAAA
M2(aa1-97) F	CACCATGAGTCTTCTAACCGAGGTCGAAACGCCTACCAGAAACGGATGGG
M2(aa1-97) R	TTACTCCAGCTCTATGTTGACAAAATG
M2 pMal F EcoRV	GATATCAGTCTTCTAACCGAGGTCGAAACGCCT
M2 pMal R BamHI	GGATCCTTACTCCAGCTCTATGTTGACAA

### Expression of recombinant M2 proteins

Complete M2 constructs were transformed into BL21 Star (DE3) One Shot cells (Invitrogen).

Cells containing Pet100 plasmid were grown in LB Broth containing 100 μg/ml ampicillin. Expression of His-M2 was induced for four hours using 0.05 mM isopropythio *b*-D-galactosidase (IPTG). Recombinant His-M2 protein was extracted from inclusion bodies and purified with the probond (Ni)-chelating resin column according the denaturating protocol provided by the manufacturer (Invitrogen).

Cells containing the pMAL-p5X plasmid were grown in LB Broth with 100 ug/ml ampicillin and 2.0 g glucose/l. Expression of MBP-M2 was induced for four hours using 0.3 mM IPTG. Maltose-binding protein (MBP) purification was performed with the Amylose resin column according to the total cell extract protocol provided by the manufacturer (New England Biolabs).

### Electrophoresis and Western blot analysis

Proteins (0.1–1.0 μg/lane) were analyzed by SDS-PAGE using 10% polyacrylamide gels. Protein bands were visualized by Gelcode Blue Stain reagent (Thermo Scientific). Proteins (0.1–1.0 μg/lane) were transferred electrophoretically from the gels onto nitrocellulose membrane and immunostained using rabbit anti-M2e (Immune Technology Corp.) followed by alkaline phosphatase-conjugated mouse anti-rabbit immunoglobulin G (IgG) (Sigma).

### Peptides and tetramers

A synthetic M2e peptide composed of amino acids 1–24, with an additional biotinylated C-terminal lysine (sequence: MSLLTEVETPTRNGWECKCSDSSDK-biotin) was obtained from GenScript (Genscript). To increase immunogenicity of this M2e peptide, a tetramer was constructed by adding the peptide to streptavidin (Applichem) in an 8:1 molecular ratio for an hour at room temperature. Unbound peptide was removed by centrifugation over a 30,000 kD Vivaspin 500 Filter (Sartorius).

A peptide representing a linear B cell epitope of the *Mycobacterium avium* subspecies *paratuberculosis* (MAP) HSP70 protein (sequence: CITDAVITVPAYFND-biotin) was used as a control [32].

### Immunization

20 chickens were divided into four groups of five animals. At day 21 of age animals were immunized in the breast muscle with a BD Plastipak syringe with a 0.45 × 10 mm needle (BD) with 150 μl of His-M2 protein vaccine or 100 μl of the other vaccines. A homologous booster immunization was given at day 42 of age. Group 1 received the subunit vaccine of 200 μg His-M2 protein. Group 2 received 40 μg of the M2e peptide. Group 3 received a vaccine consisting of the M2e-streptavidine tetrameric construct containing 40 μg M2e. Group 4 received a vaccine consisting of the HSP-streptavidin tetrameric construct containing 20 μg HSP-peptide. All vaccines were adjuvanted with Stimune (Prionics), four parts of the water phase containing the antigen were mixed with five parts Stimune (v/v). The amount of molecules present in 200 μg His-M2 protein was calculated and the amount of peptide molecules injected in groups 2, 3 and 4 was adjusted to match this amount.

Serum samples were taken before immunization, three weeks after vaccination and two weeks after booster (resp. day 21, 42 and 56 of age) by vene puncture of the wingweb vene. Chickens were sacrificed by cervical dislocation at 56 days of age; serum was collected, and stored at −20°C until analyzed.

### Antibody detection

Antibodies to the whole M2 protein or M2e peptide were detected by an indirect ELISA test. 96-wells plates were coated with 0.5 μg/well MBP-M2 protein or M2e peptide in pH 9.6 bicarbonate buffer (100 μl). Plates were blocked with 200 μl ELISA blocking buffer (Roche Diagnostic GmbH). After each incubation step, plates were washed three times with PBS-0.01% Tween20. Incubations were performed for 20 min at 37°C. Plates were incubated with 1:10 diluted chicken serum in PBS-0.05% Tween20, mouse anti chicken IgL (1:1000) (Southern Biotechnology), goat anti mouse-Ig-AP (1:1000) (DAKO) and p-nitrophenyl phosphate (PNPP) substrate (Pierce). The OD was measured using a Biorad microplate reader at 405 nm. Samples were corrected for background signal.

Antibodies recognizing virus were detected using the AI Flockcheck test (IDEXX) according to the manual of the manufacturer.

### *In vitro* infection of MDCK cells

We infected MDCK cells to test whether serum from vaccinated animals could bind native M2. Twenty-four-well plates were seeded with 5 × 10^5^ MDCK cells per well. At 80% confluence, cells were infected (or left uninfected as a control) with 200 μl infection medium (DMEM + Glutamax + 0.1% BSA) with 10^6^ egg infective doses (EID_50_) of H9N2 A/Chicken/Saudi Arabia/SP02525/3AAV/2000 per ml. Plates were incubated at 37°C for 1 h and non-bound virus was washed away with PBS. Cells were cultured overnight at 37°C in infection medium with 1.0 μg/ml trypsine (Wortington). After washing with PBS and adding 5.0 mM EDTA single cells were obtained and pooled. Pool samples were taken for flow cytometry.

### Flow cytometry

Cells were stained for 30 min with Chicken serum obtained from animals at 56 days of age, five times diluted in PBA (PBS with 0.5% BSA and 0.005% NaN_3_), mouse anti-chicken IgL (1:100)_(Southern Biotech) and goat anti-mouse FITC (1:100) (Southern Biotech). Positive controls were cells stained with rabbit polyclonal anti-M2 (1:100) or rabbit anti-HA1 (1:100) (Immune Technology Corp.) followed by donkey anti-rabbit IgG Alexa Fluor 488 (1:100) (Invitrogen); chicken anti H9N2 antiserum (1:100) (H9N2 positive chicken reference serum obtained from GD Veterinary Health Service, Deventer, The Netherlands) followed with mouse anti-chicken IgL (1:100)_(Southern Biotech). As conjugate controls, rabbit pre-immune serum (Immune Technology Corp.) and negative chicken serum were used.

2.0 μl 7-Aminoactinomycin D (7-AAD) was added to all samples and after 1 min cells were washed and fixed in 2% paraformaldehyde. A total of at least 20,000 events was acquired using a FACScan flowcytometer (BD) and data were analysed using the FlowJO software (Threestar Inc.).

### Statistics

Data were analysed with ANOVA using Graphpad Prism. If variances were not equal data were log transformed or a Kruskal-Wallis test was performed. A Bonferroni or Dunn post hoc test was used and the level of significance was set at p < 0.05.

## Abbreviations

Ab: Antibody; AIV: Avian influenza virus; HA: Hemagglutinin; HBc: Hepatitis B virus core; HPAI: Highly pathogenic avian influenza virus; LPAI: Low pathogenic avian influenza virus M1: matrix protein 1; M2: Matrix protein; M2e: Extracellular part of the M2 protein; NA: Neuraminidase; NDV: Newcastle disease virus; SIV: Swine influenza virus; vRNP: Viral ribonucleoprotein.

## Competing interests

The authors declare that they have no competing interests.

## Authors’ contributions

WS carried out most of the experiments and wrote the manuscript. JH and RS made the M2 protein constructs and JH helped with the ELISA experiments, Electrophoresis and Western Blots. LV and AK conceived the study, participated in its design and coordination and also helped to look over the manuscript. All Authors read and approved the final manuscript.
